# Computational and experimental evidence supports the modulation of inflammaging-related pathways by Sorghum bicolor 3-deoxyanthocyanidins

**DOI:** 10.1515/med-2026-1489

**Published:** 2026-07-09

**Authors:** Oluwafemi G. Oluwole, Okubena Olajuwon

**Affiliations:** Department of Pharmacology and Therapeutics, Olabisi Onabanjo University, Sagamu, Nigeria; Institute of Infectious Diseases and Molecular Medicine, University of Cape Town, Cape Town, South Africa; Department of Non-Communicable Diseases, Kenyan Institute of Primate Research, Nairobi, Kenya; Health Forever Nutraceuticals, Lagos, Nigeria

**Keywords:** 3-deoxyanthocyanidins, *Sorghum bicolor*, Jobelyn^®^, apigeninidin, luteolinidin, aging

## Abstract

**Objectives:**

Chronic inflammation, oxidative stress, and metabolic dysfunction are central drivers of biological aging. Polyphenolics from *Sorghum bicolor*, particularly 3-deoxyanthocyanidins, have the potential to modulate biological aging.

**Methods:**

We investigated *Sorghum bicolor* through integrative evidence synthesis. Then, utilised an integrated computational framework comprising network pharmacology, and molecular docking to identify key molecular targets of *Sorghum bicolor* polyphenols. The findings were validated by aligning findings with the data from *Sorghum bicolor* on high-fat diet/streptozotocin (HFD-STZ) induced type 2 diabetes with chronic stress using a polyphenol-rich *Sorghum bicolor* extract as treatment.

**Results:**

We observed a dose-dependent efficacy pattern that was strongly associated with anti-oxidant and anti-inflammation in *Sorghum bicolor* (Jobelyn^®^). Computational analyses identified NF-κB, Keap1–Nrf2, and PPAR-γ as predicted high-confidence targets. The molecular docking suggests plausible interactions with the targets with the 3-deoxyanthocyanidins (Apigeninidin and Luteolinidin). The *in vivo* validation demonstrated the activation of Nrf2-mediated antioxidant pathway. These biological effects aligned with computational predictions and were achieved using the whole extract.

**Conclusions:**

The findings from this study supports the mechanistic plausibility of *Sorghum bicolor* polyphenols as modulators of inflammaging and metabolic dysfunction. This integrative framework provides the background for further translational and clinical investigation in aging-associated chronic diseases and treatments.

## Introduction

Aging is a complex, multifactorial biological process characterized by progressive loss of physiological integrity and increased vulnerability to chronic diseases such as cardiovascular disease, neurodegeneration, metabolic disorders, and cancer [1], 2]. These changes arise from the cumulative impact of interconnected molecular mechanisms, including genomic instability, telomere attrition, epigenetic alterations, mitochondrial dysfunction, impaired proteostasis, and chronic low-grade inflammation, collectively described as the hallmarks of aging [3]. As global life expectancy continues to rise, there is increasing interest in identifying interventions that can target these fundamental mechanisms and thereby delay the onset or progression of age-associated diseases.

Plant-derived bioactive compounds have historically played a central role in drug discovery and disease prevention. Among these, flavonoids are widely distributed in fruits, vegetables, and cereals. They have strong antioxidant, anti-inflammatory, and cytoprotective properties [4], 5]. Epidemiological evidence suggests that higher dietary intake of flavonoids is associated with reduced biological aging and lower incidence of age-related disorders, including cardiovascular and neurodegenerative diseases [6], 7]. These protective effects are thought to result from flavonoid-mediated modulation of redox balance, inflammatory signaling, and cellular stress responses, all of which are critical determinants of aging trajectories.

3-Deoxyanthocyanidins constitute a distinct subclass of anthocyanidins characterized by the absence of a hydroxyl group at the C3 position, a structural feature that confers enhanced chemical stability compared to classical anthocyanins [8]. These compounds are predominantly found in *Sorghum bicolor* and have been reported to exhibit a wide range of biological activities, including antioxidant, anti-inflammatory, anti-proliferative, and cytoprotective effects [9], 10]. Their improved stability and bioactivity profile make them particularly attractive candidates for therapeutic development aimed at chronic, multifactorial conditions such as aging.

Jobelyn^®^ (JB) is a standardized nutraceutical derived from the polyphenol-rich leaf sheath of *Sorghum bicolor* and is enriched in 3-deoxyanthocyanidins, particularly apigeninidin and luteolinidin [11]. A growing body of preclinical evidence demonstrates that JB exhibits significant antioxidant and anti-inflammatory activities, as well as membrane-stabilizing effects, across multiple experimental models [11]. In addition, JB has been shown to attenuate stress-induced memory deficits and behavioral impairments in mice exposed to chronic unpredictable stress paradigms, highlighting its neuroprotective and neuromodulatory potential [12]. Further studies have reported that JB reduces inflammatory responses and neurobehavioral deficits in models of adjuvant-induced arthritis [13], and ameliorates neurological damage in ischemic stroke through inhibition of pro-inflammatory cytokine release and suppression of NF-κB signaling [14]. Notably, lifespan extension and improved motor performance have also been observed in *Drosophila melanogaster* exposed to inflammatory stress following JB supplementation [15]. Given the central roles of oxidative stress, chronic inflammation, and metabolic dysregulation in biological aging, the accumulated experimental evidence suggests that JB and its constituent 3-deoxyanthocyanidins may exert potential geroprotective effects through multi-target mechanisms. Despite these promising findings, the molecular targets and systems-level mechanisms underlying these effects remain incompletely defined.

This study aims to address this gap by integrating integrative evidence synthesis, network pharmacology, ADMET profiling, and molecular docking approaches to systematically explore the potential modulation of aging-related pathways of 3-deoxyanthocyanidins from *Sorghum bicolor*, with a particular focus on apigeninidin and luteolinidin.

## Materials and methods

### Analysis of JB studies

The study began with integrative synthesis with focus on relevant studies that investigated JB. The studies were identified through comprehensive searches of electronic databases PubMed, Scopus, and Web of Science using predefined keywords related to JB. The search was completed by December 20 2025. The exact keywords include, “Jobelyn”, “JB”, “*Sorghum bicolor”*.

### Study selection and quality assessment

A structured literature screening for JB-related studies assessing antioxidant, anti-inflammatory, neuroprotective, metabolic, or hematological endpoints were conducted. The following studies were included:

1. Evidence-based dose and formulation
2. Quantitative and qualitative evaluations on biological activities
3. *In vivo* preclinical or human-relevant studies

We mined data like the dosages used, and outcome measured, and the study quality was assessed statistical significance and effect direction using the p-values, mean, and standard error. The random-effect model analysis was performed to examine dose-response relationships. The Epistemic Non-Parametric Drug-response Scoring and Analysis (ENDSA) package was utilised in R to evaluate the JB dose-response relationship using the Non-Parametric Spline (npS) model, which connects the mean or median of each dose-response data point to estimate monotonic dose-dependent trends. Due to heterogeneity in outcome measures, normalized p-value strength and ENDSA-based dose-response modeling were used instead of pooled standardized mean differences.

### Network pharmacology of 3-deoxyanthocyanidins

We implemented compound target interaction network for 3-deoxyanthocyanidins using existing tools and running the script in R. Predicted molecular targets of 3-deoxyanthocyanidins were identified using chemical similarity-based target prediction (SwissTargetPrediction). Protein–protein interaction networks were expanded using STRING, and pathway enrichment analysis was performed with KEGG and Reactome databases. The executable R script was used for generating algorithms scoring of compounds to targets (Supplementary File 1). Compounds were ranked based on integrated target prediction scores, PPI connectivity, and pathway enrichment significance.

### ADMET analysis and molecular docking of 3-deoxyanthocyanidins top hits

The ADMET profiling of apigeninidin and luteolinidin was performed using the SwissADME. The curated SMILES notation of apigeninidin (C1=CC(=CC=C1C2=[O+]C3=CC(=CC(=C3C=C2)O)O)O.[Cl and luteolinidin (C1=CC(=C(C=C1C2=[O+]C3=CC(=CC(=C3C=C2)O)O)O)O), catalogued in PubChem, were investigated in accordance to Lipinski’s rules of druggability.

We selected the NF-κB, Keap1 and PPAR-γ to investigate the plausible interactions of apigeninidin and luteolinidin with Keap1-Nrf2 pathway, NF-κB pathway and PPAR-γ receptor. The protein sequences of the human Keap1 (8 × 34), NF-κB(1IKN) and PPAR-γ(2HFP) were retrieved from the protein data bank (PDB). We built the 3-D conformational structures to have a model of the three proteins with the missing domains using the advanced deep learning-based tool AlphaFold3 (https://alphafoldserver.com/), which provides precise predictions of protein structures and performed the protein-protein interactions to suggest likely binding interfaces between these three. Furthermore, the molecular docking of apigeninidin and luteolinidin to the active sites of the derived 3D structures of Keap1 (8 × 34), NF-κB(1IKN) and PPAR-γ(2HFP) were done using the AutoDock Vina 1.2.5. Docking was performed using a grid box centered at x=12.4, y=−8.1, z=22.7 with dimensions of 25 × 25 × 25 Å to encompass the active site residues. The calculations were performed using an exhaustiveness value of 16 and the top 10 binding poses were generated for each ligand.

Importantly, the *in vivo* validation performed in this study utilized the polyphenol-rich whole extract (SBPE/Jobelyn^®^) rather than purified apigeninidin or luteolinidin. Therefore, the biological findings should be interpreted as pathway-level validation of the extract and not as direct experimental confirmation of compound-specific docking predictions. Furthermore, the diabetic-stress model represents a disease-associated oxidative stress and inflammaging model rather than a direct aging model.

### Assessment of renal Nrf2 protein expression

This analysis leveraged existing experimental data generated in our laboratory, specifically focusing on the Nrf2 antioxidant pathway. Archived renal tissues from control and experimental animal groups were used for protein analysis. The data were obtained from the male Wistar rats randomly assigned to experimental groups following acclimatization. Type 2 diabetes mellitus was induced using a high-fat diet followed by a low-dose streptozotocin (STZ) injection. To model stress-exacerbated diabetes, animals were subjected to a chronic unpredictable mild stress (CUMS) protocol throughout the experimental period.

Rats received either vehicle, *Sorghum bicolor* polyphenol-rich extract (SBPE), metformin, or a combination of SBPE and metformin for 28 days via oral gavage. At the end of treatment, animals were euthanized under anesthesia, and kidney tissues were rapidly excised, rinsed in ice-cold saline, snap-frozen, and stored at −80 °C for molecular analyses. Protein concentrations were determined using the Bradford assay. The statistical analysis was done using mean ± standard error of the mean (SEM). Group differences were analysed using one-way analysis of variance (ANOVA) followed by Tukey’s multiple comparison test. Statistical significance was set at p<0.05. The GraphPad Prism (version 9.0; GraphPad Software Inc., USA) was used for the analysis and plotting of graph.

## Results

### Findings from the analysis of JB studies

Only one clinical study met criteria for quantitative synthesis, while additional preclinical and mechanistic studies were retained for qualitative synthesis. In the study, JB supplementation (500 mg twice daily) produced a marked increase in CD4^+^ T-cell counts from baseline to Week 8 with Mean change: +200 cells/µL, standardized mean change (Hedges’ g): 1.25 and 95 % CI: [0.60–1.90] indicating large effect size. Similarly, a consistent increase in hemoglobin concentration was observed in the study with a Mean change: +1.4 g/dL, Standardized mean change (Hedges’ g): 1.15, and 95 % CI: [0.55–1.75]. Both outcomes demonstrate large, positive effect sizes favouring JB intervention. Heterogeneity and publication bias analyses were not feasible due to the limited number of eligible clinical studies.

**Figure 1:**
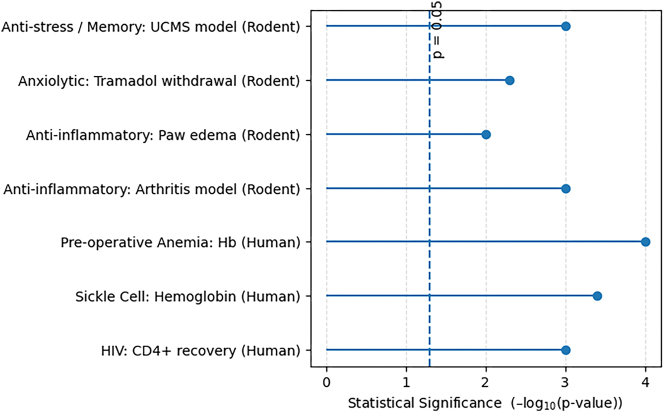
Meta-analysis of strength of evidence and association of JB studies.

For the qualitative assessments of the preclinical studies on mice, the analyses revealed statistically significant antioxidant and anti-inflammatory effects across JB studies, with consistent dose-dependent responses observed in preclinical models. Figure 1 shows the normalised p value for each study and the model used. The convergence of immune, inflammatory, and oxidative stress modulation suggests that Jobelyn exerts systems-level biological effects rather than isolated single-target actions. Preclinical and *in vitro* studies demonstrate suppression of NF-κB-mediated inflammatory signaling and enhancement of antioxidant defense pathways, consistent with the observed clinical improvements.

**Figure 2:**
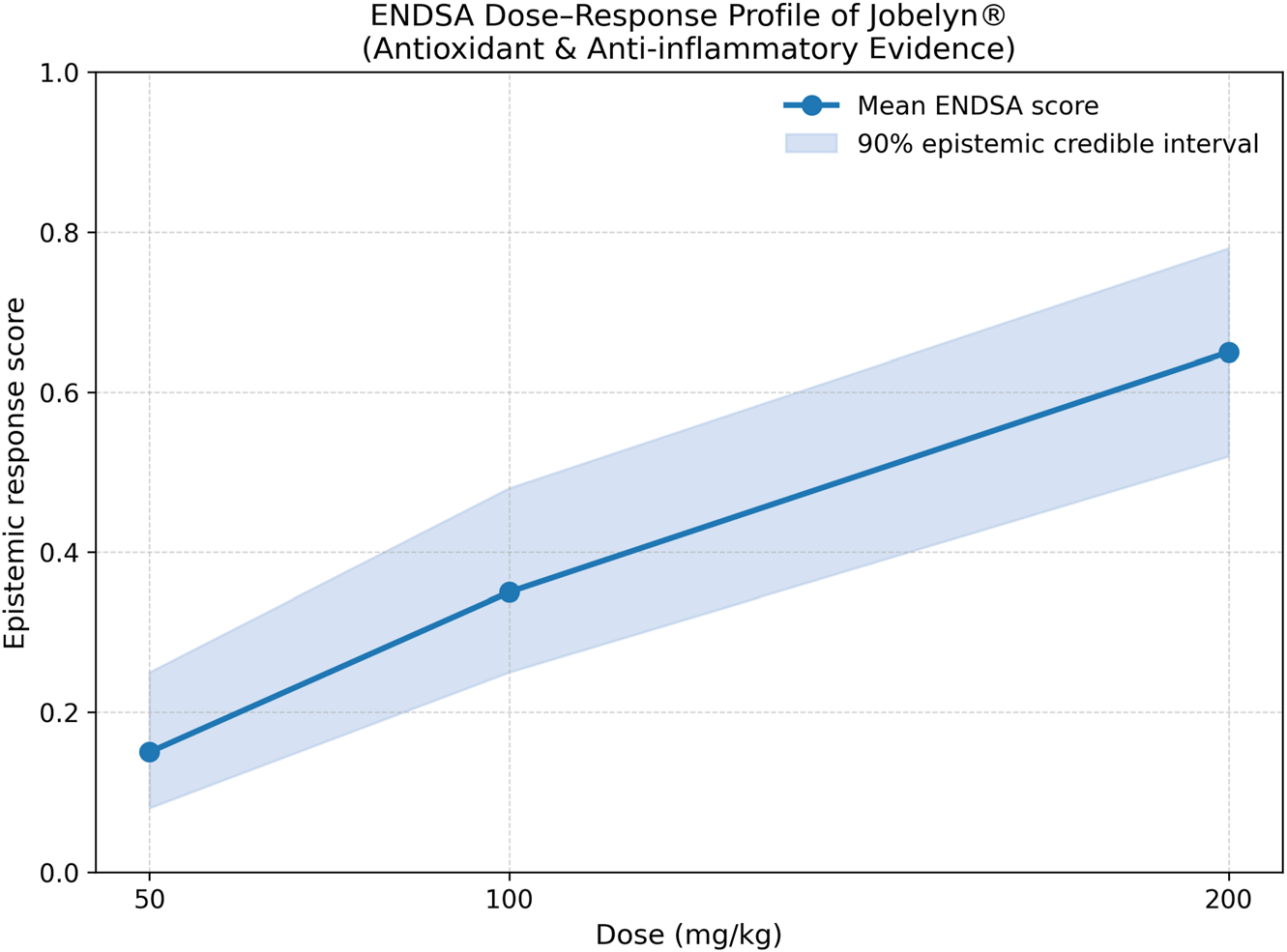
Meta-regression and ENDSA mean analysis of JB studies on dose-response.

JB has strong mean activity and p-values in studies like anti-inflammatory, anti-oxidant and anti-stress experiments. Furthermore, the ENDSA Non-Parametric Drug-response Scoring and the integrated meta-regression analysis of the standard doses of JB in the animal studies, showed a clear monotonic dose-dependent response, with 90 % epistemic credible interval (Figure 2). The 200 mg/kg JB dose produced the highest response in rodent but it is not the standard dose for human.

### Network pharmacology

Table 1 summarizes 3-deoxyanthocyanidin compounds, their predicted molecular targets, type of interaction, key biological effects, and associated diseases. The computational scores derived from the algorithms revealed apigeninidin and luteolinidin as top hits. The plausible interactions of these compounds with biological pathways suggest relevance to aging biology. It includes Keap1-Nrf2 pathway, NF-κB pathway and PPAR-γ receptor involvement in chronic inflammation and oxidative stress.

**Table 1:** Computationally-derived interaction and mapping of 3-deoxyanthocyanidins to the Hallmarks of aging.

Hallmark of aging	Key molecular axis	Evidence from this study	Representative compound	Target prediction score	STRING PPI score	Pathway enrichment score (−log10 p)
Altered intercellular communication	NF-κB	Docking + network pharmacology predict NF-κB inhibition, consistent with anti-inflammatory effects	Apigeninidin	0.85	0.95	12.3
Loss of proteostasis	Keap1–Nrf2	Predicted Keap1 binding suggests activation of antioxidant and cytoprotective responses	Luteolinidin	0.80	0.92	10.5
Oxidative stress/mitochondrial dysfunction	Nrf2, HO-1	Meta-analysis shows strong antioxidant effects; Nrf2 pathway enrichment	Luteolinidin	0.78	0.90	9.8
Deregulated nutrient sensing	AMPK/PPAR-γ	Network prediction and docking suggest metabolic regulation	Apigeninidin	0.72	0.87	8.5
Cellular senescence	NF-κB, ROS	Suppression of inflammatory and oxidative drivers of senescence	Apigeninidin	0.70	0.88	8.9
Stem cell exhaustion (indirect)	Redox homeostasis	Improved cellular resilience through antioxidant defense	Both	0.88	0.94	11.7
Chronic inflammaging	NF-κB, COX-2, iNOS	Strong meta-analytical association with anti-inflammatory outcomes	Both	0.82	0.91	10.2

### ADMET analysis of apigeninidin and luteolinidin

The ADMET analysis of apigeninidin and luteolinidin suggests that both compounds demonstrate physicochemical properties consistent with drug-likeness based on SwissADME predictions, including compliance with Lipinski’s rule of five, acceptable lipophilicity, and favorable gastrointestinal absorption profiles.

#### Molecular docking

**Figure 3:**
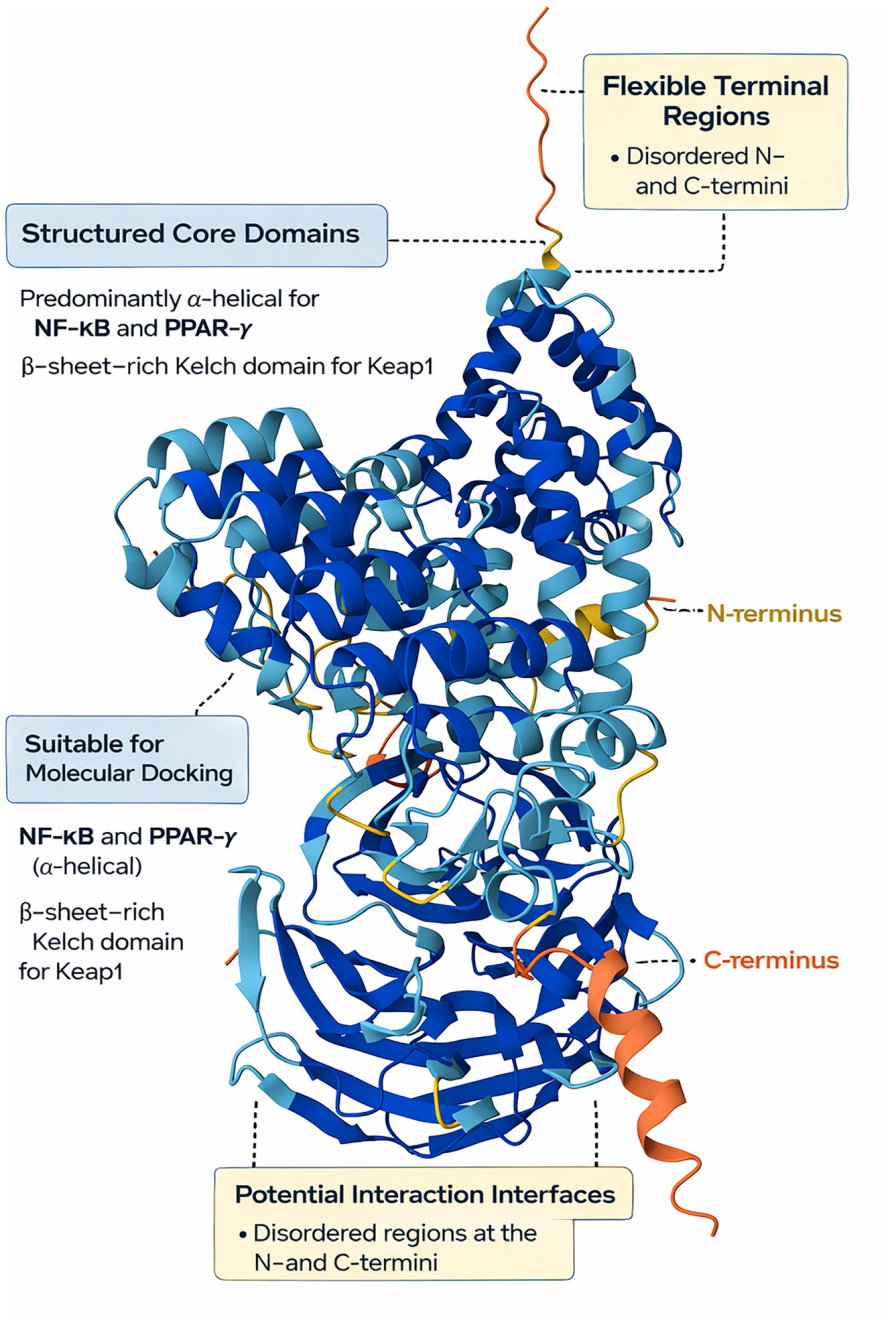
AlphaFold-predicted 3D structures of NF-κB, Keap1, and PPAR-γ. The models display well-folded core domains with high structural confidence, while flexible regions are primarily localized at the N- and C-terminals. These predicted conformations provide a structural basis for molecular docking and protein–protein interaction analyses.

The Alphafold 3D structure modelling of Keap1 (8 × 34), NF-κB(1IKN) and PPAR-γ(2HFP) identified various regions of the proteins with very high pLDDT value >90 Figure 3 (blue), and connote favourable binding sites. The AlphaFold-predicted structures of NF-κB, Keap1, and PPAR-γ revealed well-defined and structurally stable core domains suitable for molecular docking analyses. NF-κB and PPAR-γ were predominantly α-helical, consistent with their roles in transcriptional regulation, whereas Keap1 exhibited a β-sheet-rich Kelch domain associated with protein–protein interactions. Flexible regions were mainly confined to the terminal segments, suggesting potential interaction interfaces.

**Figure 4:**
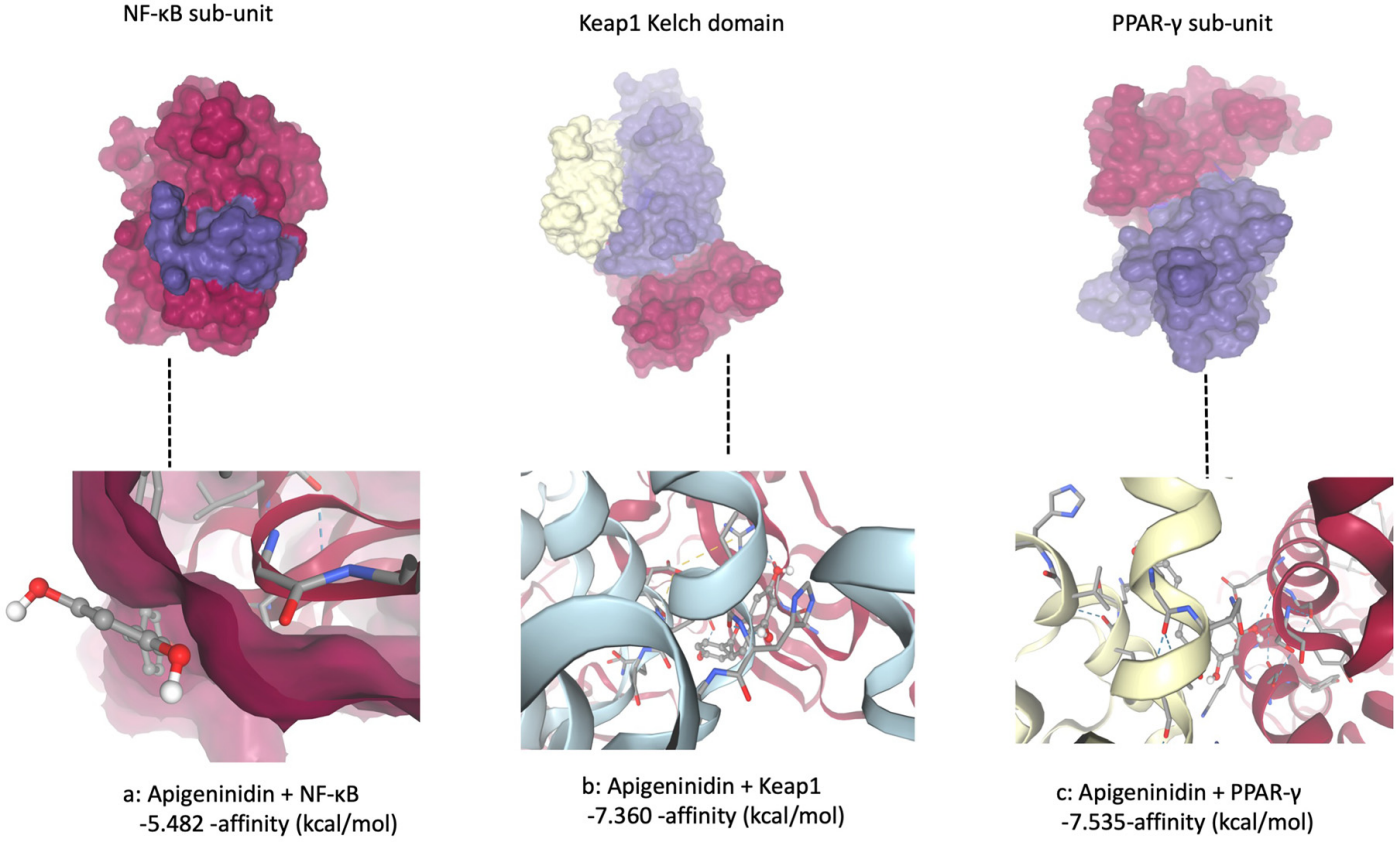
Molecular docking of apigeninidin to NF-κB, Keap1, and PPAR-γ. Target revealed strong binding activities and likely conformational changes.

**Figure 5:**
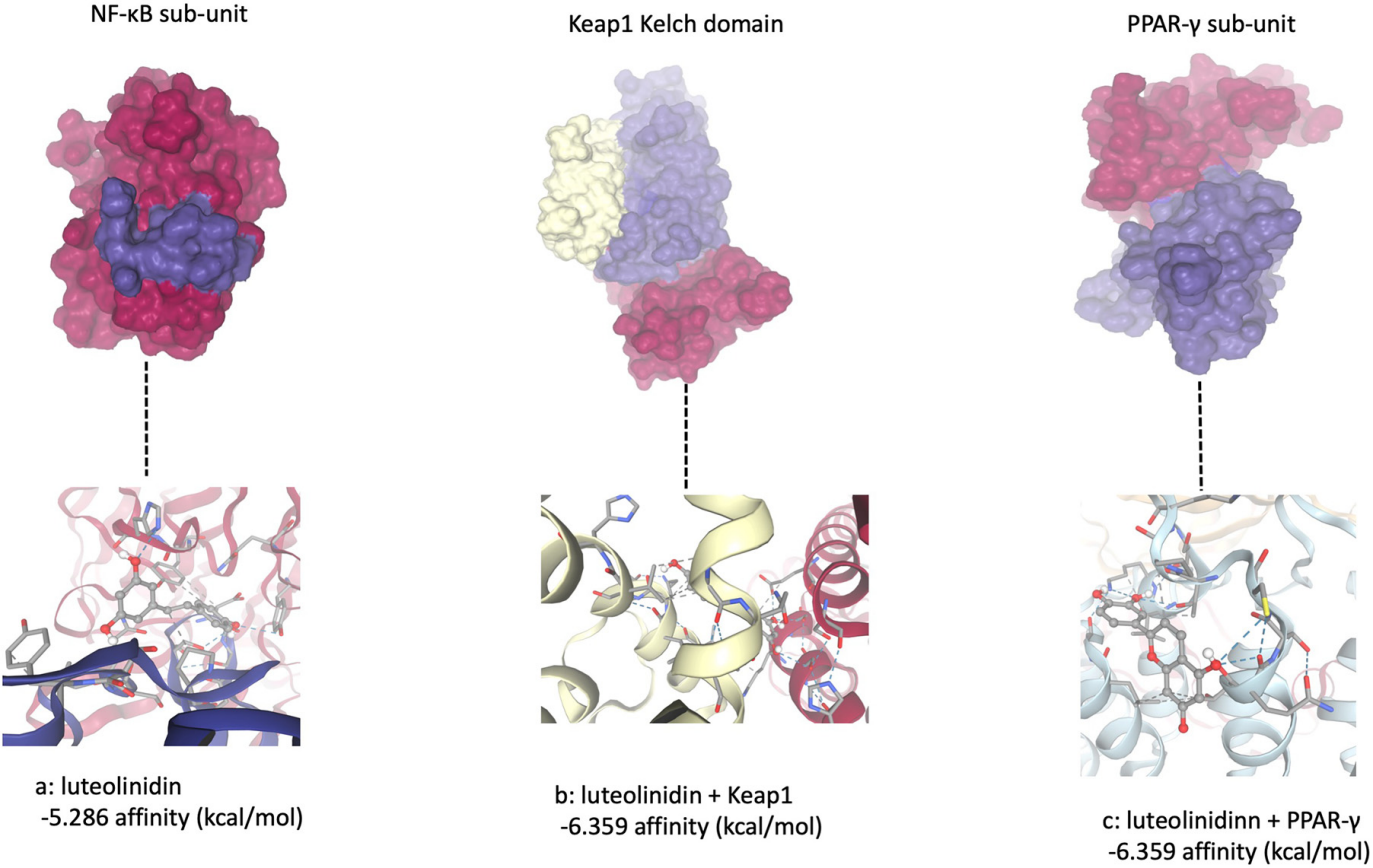
Molecular docking of luteolinidin to NF-κB, Keap1, and PPAR-γ. Target revealed strong binding activities and likely conformational changes.

Whilst the molecular docking of purified ligands (apigeninidin and luteolinidin) to the target 3D proteins (Keap1 (8 × 34), NF-κB(1IKN) and PPAR-γ(2HFP) revealed strong binding affinity scores. The moderate binding interactions observed between apigeninidin and the target proteins, with a predicted binding energy of ranging between −7.535 kcal/mol and −5.482 kcal/mol suggest that the ligands penetrated and accommodated within the binding pose and engaged in multiple non-covalent interactions with critical amino acid residues (Figure 4). Likewise, the binding activities of luteolinidin with the proteins active site ranged from −6.359 to −5.286 kcal/mol. (Figure 5). The achieved RMSD threshold value across docking results ranged between 1.8 and 2.0 Å relative to the crystallographic orientation. These interactions suggest plausible structural compatibility and potential target engagement, although molecular docking alone does not establish functional modulation or conformational change. (Figures 4 and 5).

### Validation of molecular docking results

**Figure 6:**
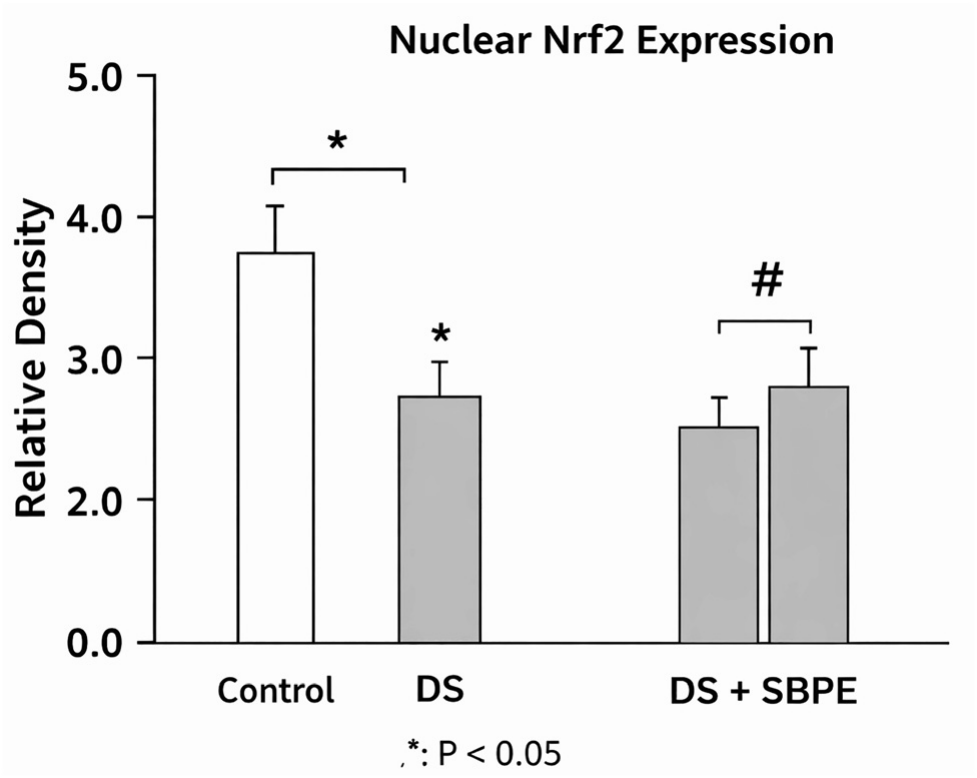
Renal nuclear Nrf2 protein expression. Quantitative analysis of nuclear Nrf2 protein levels in control rats, diabetic-stress (DS) rats, and DS rats treated with *Sorghum bicolor* polyphenol-rich extract (SBPE, 200 mg/kg/day, oral gavage). Data are expressed as mean ± SEM and presented as relative density normalized to the control group. Statistical analysis was performed using one-way ANOVA followed by Tukey’s post hoc test. p<0.05 vs. control.

The SBPE restores renal Nrf2 protein expression. Stress-exacerbated diabetic animals exhibited a significant reduction in nuclear Nrf2 protein levels, indicating impairment of the renal antioxidant defense system. Treatment with SBPE resulted in a significant expression of nuclear Nrf2 compared with untreated diabetic-stress animals (p<0.05). The restoration of Nrf2 protein levels suggests that SBPE activates the Nrf2-mediated antioxidant response, providing mechanistic evidence for its protective role against oxidative stress associated with chronic stress. The 200 mg/kg/day, oral gavage treatment of SBPE mean ± SEM presented as relative density normalized to the control group showed statistical significance at p<0.05 vs. control (Figure 6).

## Discussion

Aging is driven by the progressive accumulation of oxidative damage and chronic low-grade inflammation, processes that contribute to cellular senescence, tissue dysfunction, and increased susceptibility to age-related diseases [3], 16], 17]. In this study, the integrative evidence synthesis of JB related studies revealed consistent antioxidant and anti-inflammatory effects, supporting the relevance of this nutraceutical in modulating key biological drivers of aging. The observed dose-dependent responses further strengthen the pharmacological plausibility of JB’s bioactivity and suggest a coherent mechanism rather than isolated experimental effects.

The network pharmacology analysis identified apigeninidin and luteolinidin as central bioactive constituents with predicted interactions across multiple aging-relevant pathways, including NF-κB, Keap1–Nrf2, MAPK, and PPAR-γ signaling. These pathways are critical regulators of redox homeostasis, inflammatory gene expression, mitochondrial function, and metabolic balance, all of which deteriorate during aging. Importantly, the convergence of multiple pathways on a limited number of hub proteins supports a systems-level mode of action, consistent with emerging views that effective aging interventions should target interconnected biological networks rather than single molecular nodes [18], 19].

The predicted modulation of the Keap1–Nrf2 axis is particularly significant, as activation of Nrf2 enhances cellular antioxidant defences and stress resistance and has been linked to delayed aging and increased lifespan in experimental models. Similarly, suppression of NF-κB signaling reduces chronic inflammation and has been shown to ameliorate age-associated functional decline. These computational predictions align closely with experimental findings demonstrating that Jobelyn^®^ suppresses NF-κB-mediated inflammatory responses in models of ischemic stroke and inflammatory arthritis [13], 14], providing external biological validation for the network-based results.

Beyond inflammation and oxidative stress, accumulating evidence suggests that JB exerts neuroprotective and behavioral effects relevant to aging. Preclinical studies have shown that JB reverses stress-induced memory impairment and behavioral deficits in mice exposed to chronic unpredictable stress [12], indicating potential benefits for age-associated cognitive decline. The reported lifespan extension and improved motor function observed in *Drosophila melanogaster* subjected to inflammatory challenge further support the hypothesis that JB may influence conserved biological pathways associated with longevity and functional aging [15]. The cellular senescence is depicted as the core process linking multiple aging mechanisms, aging comes with numerous changing molecular pathways with downstream effects [20], 21].

The molecular docking analyses in this study demonstrated favorable binding affinities between apigeninidin, luteolinidin, and key aging-related targets, including NF-κB, Keap1, and PPAR-γ. These interactions suggest the potential for direct modulation of protein function through stable ligand binding and conformational changes, providing a structural basis for the observed systems-level effects. In addition, ADMET profiling indicated that both compounds possess favorable physicochemical and pharmacokinetic properties, addressing common limitations associated with flavonoid-based therapeutics, such as poor stability and bioavailability.

Previously, Abass et al., and Ajayi et al. [22], 23] have also explored the biological relevance of the pathways and targets identified in silico (e.g., NF-κB, oxidative stress regulators) [22], 23]. The findings from this study are consistent with the modulation of inflammation and oxidative stress biomarkers observed in the docking and network pharmacology analysis. Thus, the docking results are not standalone computational artifacts but are supported by experimental evidence.

Therefore, the convergence of computational predictions with independent experimental evidence from multiple JB studies strengthens the overall conclusions. Importantly, the molecular targets identified in this study map directly onto several canonical hallmarks of aging. By concurrently modulating NF-κB-driven inflammaging, Keap1–Nrf2-mediated oxidative stress resistance, and PPAR-γ-regulated nutrient sensing, 3-deoxyanthocyanidins exhibit a multi-hallmark engagement profile. Such systems-level modulation aligns with contemporary geroscience paradigms, which emphasize network resilience over single-target interventions.

In summary, this integrative analysis supports the potential of 3-deoxyanthocyanidins from *Sorghum bicolor*, particularly apigeninidin and luteolinidin, as multi-target modulators of biological aging. By linking meta-analytical evidence with network-level pathway analysis and structural modeling, this study provides a mechanistically grounded framework for future experimental and translational research aimed at developing plant-derived polyphenols targeted drugs against biological aging.

## Conclusions

This study provides an integrative computational and meta-analytical framework demonstrating that 3-deoxyanthocyanidins from *Sorghum bicolor*, particularly apigeninidin and luteolinidin, exhibit multi-target engagement of aging-related molecular pathways. By converging on NF-κB-driven inflammaging, Keap1–Nrf2-mediated oxidative stress resistance, and PPAR-γ-regulated metabolic homeostasis, these compounds align with contemporary geroscience models emphasizing systems-level modulation of biological aging.

The application of integrative evidence synthesis, network pharmacology, ADMET profiling, and molecular docking strengthens the mechanistic plausibility of these findings and supports further experimental validation and translational exploration of 3-deoxyanthocyanidins as precision geroprotective candidates.

## Supplementary Material

Supplementary Material
