# Supplementary material for: Computational and experimental evidence supports the modulation of inflammaging-related pathways by Sorghum bicolor 3-deoxyanthocyanidins
Source: Open Med (Wars). 2026 Jul 9;21(1):20261489. doi: 10.1515/med-2026-1489 (PMC13351292; doi:10.1515/med-2026-1489)
Supplement: Supplementary file 1 — Supplementary Material [file j_med-2026-1489_suppl_001.docx]

library(clusterProfiler)

library(org.Hs.eg.db)

library(STRINGdb)

library(dplyr)

# -------------------------------

# Step 1: Load predicted targets

# -------------------------------

# Target list for 3-deoxyanthocyanidins (gene symbols)

targets <- c("NFKB1","PTGS2","NOS2","AKT1","MAPK1","BCL2","TP53")

# -------------------------------

# Step 2: Map gene symbols to Entrez IDs

# -------------------------------

entrez_ids <- bitr(targets, fromType="SYMBOL",

toType="ENTREZID", OrgDb=org.Hs.eg.db)

entrez_ids

# -------------------------------

# Step 3: STRING network expansion

# -------------------------------

string_db <- STRINGdb$new(version="11.5", species=9606, score_threshold=400)

mapped_targets <- string_db$map(entrez_ids, "ENTREZID", removeUnmappedRows = TRUE)

string_network <- string_db$get_interactions(mapped_targets$STRING_id)

head(string_network)

# -------------------------------

# Step 4: KEGG pathway enrichment

# -------------------------------

kegg_enrich <- enrichKEGG(gene = mapped_targets$ENTREZID,

organism = 'hsa',

pvalueCutoff = 0.05)

head(kegg_enrich)

# -------------------------------

# Step 5: Reactome pathway enrichment

# -------------------------------

reactome_enrich <- enrichPathway(gene = mapped_targets$ENTREZID,

organism = "human",

pvalueCutoff = 0.05)

head(reactome_enrich)

# -------------------------------

# Step 6: Output results

# -------------------------------

write.csv(kegg_enrich, "KEGG_enrichment_results.csv", row.names=FALSE)

write.csv(reactome_enrich, "Reactome_enrichment_results.csv", row.names=FALSE)

write.csv(string_network, "STRING_network_edges.csv", row.names=FALSE)
